# Commercially Available Fluoride-Releasing Restorative Materials: A Review and a Proposal for Classification

**DOI:** 10.3390/ma13102313

**Published:** 2020-05-18

**Authors:** Philippe Francois, Vincent Fouquet, Jean-Pierre Attal, Elisabeth Dursun

**Affiliations:** 1 Innovative Dental Materials and Interfaces Research Unit (URB2i, UR4462), Faculty of Health, Paris University, 1 rue Maurice Arnoux, 92120 Montrouge, France; philippe.francois@parisdescartes.fr (P.F.); vincent.fouquet@parisdescartes.fr (V.F.); jean-pierre.attal@parisdescartes.fr (J.-P.A.); 2 Bretonneau Hospital, 23 rue Joseph de Maistre, 75018 Paris, France; 3 Louis Mourier Hospital, 178 rue des Renouillers, 92700 Colombes, France; 4 Charles Foix Hospital, 7 avenue de la République, 94200 Ivry-sur-Seine, France; 5 Henri Mondor Hospital, 1 rue Gustave Eiffel, 94000 Créteil, France

**Keywords:** GIC, RM-GIC, HV-GIC, compomer, giomer, nanoionomer, Activa BioActive Restorative, Surefil One, Cention N, bioactive materials

## Abstract

Resin composite and glass ionomer cement (GIC) are the most commonly used dental materials to perform direct restorations. Both have specific characteristics that explain their popularity and their limits. More than 20 years ago, the first attempt (followed by others) to combine the advantages of these two families was performed with compomers, but it was not very successful. Recently, new formulations (also called ‘smart materials’) with claimed ion release properties have been proposed under different family names, but there are few studies on them and explanations of their chemistries. This comprehensive review aims to gather the compositions; the setting reactions; the mechanical, self-adhesive, and potential bulk-fill properties; and the ion release abilities of the large existing families of fluoride-releasing restorative materials and the new restorative materials to precisely describe their characteristics, their eventual bioactivities, and classify them for an improved understanding of these materials. Based on this work, the whole GIC family, including resin-modified and highly viscous formulations, was found to be bioactive. Cention N (Ivoclar Vivadent, AG, Schaan, Lietschentein) is the first commercially available bioactive resin composite.

## 1. Introduction

A trend regarding the development of new hybrid restorative materials that combine resin composites and glass ionomer cements (GICs) has been observed not only in the scientific literature but also in the dental industry. The approach involves combining the advantageous properties of adhesive–resin couple composites (mechanical strength, esthetics, and high bond strength) and GICs (self-adhesive properties, moisture tolerance, and ion release) [1]. This almost chimeric intention already resulted in the resinous and still self-adhesive variant of conventional GICs, namely resin-modified glass ionomer cements (RM-GICs), as well as ion releasing but still non-adhesive variants of resin composites, namely compomers and giomers [2], approximately 20 years ago. RM-GICs have been extensively tested in recent research and showed both good mechanical characteristics [3] and acceptable bond strength values [4].

These hybrid products, with their well-studied and well-described chemistry, have achieved variable clinical success, but their use has decreased in daily practice due to their limitations compared to the performance of adhesive-composite resin couples and recent generation of high-viscosity glass ionomer cements (HV-GICs) [5].

Recently, three new hybrid composites that claim to release fluoride ions were introduced to the market: Activa BioActive Restorative (Pulpdent Corporation, Watertown, MA, USA), Cention N (Ivoclar-Vivadent, AG, Schaan, Liechtenstein), and Surefil One (Dentsply-Sirona, Konstanz, Germany). Some of these materials, like those mentioned above, are sometimes called bioactive due to their ionic release, although the use of this term is controversial. It seems that only a “restorative material capable of inducing bio-remineralization by means of a sufficiently significant ionic release” can be considered bioactive [6].

The aim of this article is to provide a review of the chemical reactions responsible for the initial setting and ion release of these new formulations. To better understand their chemical and physical properties, the release capacities and indications of the different generations of GICs, compomers, and giomers are described, leading to a classification proposal based on the composition and setting reaction of these new materials.

**Figure 1:**
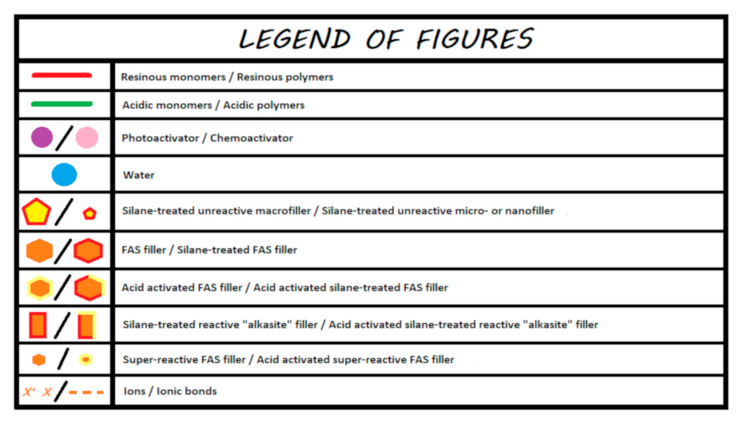
Legends for the illustrations presented in the following figures.

For the sake of simplification, reactive fillers (i.e., those activated by acids), whose compositions vary according to the manufacturer, are called fluoro-alumino-silicate (FAS) fillers. These different compositions likely produce different ion release behaviors. In the proposed diagrams, only the releases of calcium, aluminum, and fluoride ions are represented. Figure 1 illustrates the legend that contains a proposal to describe the chemistry of all the materials discussed in this review.

## 2. Conventional GICs and Their Evolution: HV-GICs and RM-GICs

From a semantic point of view and in relation to ISO standards, GICs should be called polyalkenoate cements [7]. However, the term GIC that was used by inventors [8] is commonly used and accepted in the scientific literature [9].

### 2.1. Conventional GICs

#### 2.1.1. Composition and Chemical Reactions

Conventional GICs, meaning those in their original classical form, are produced by an acid–base reaction from a powder–liquid mixture [8]. These are complex materials [10] with widely varying compositions from one formulation to another [11].

The liquid, in addition to containing water, consists mainly of polyacrylic acid [8]. Other polyacids—such as tartaric, itaconic, maleic, or tricarballylic acid—may also be added or even replaced by polyacrylic acid to modulate the reaction or the rheological properties of the material [12,13]. Schematically, the liquid is an aqueous solution containing a polyacrylic acid with numerous ionized carboxyl functional –COOH groups, which are therefore in the form of COO–. However, some formulations place the acid groups in lyophilized form directly into the powder. These groups are secondarily activated by the liquid, which almost exclusively contains water [14].

The powder contains basic reactive FAS fillers. However, this name is simplistic because, depending on the composition chosen by the manufacturer, other elements may be incorporated, such as strontium, phosphate, zinc, calcium, or sodium [11,15,16]. In addition to playing a major role in the acid–base reaction that sets the material, these FAS fillers play a role in the final mechanical properties of the material. To preserve their reactivity and given the absence of a resin in their formulation, the FAS fillers used in conventional GICs on the market are non-silanated.

**Figure 2:**
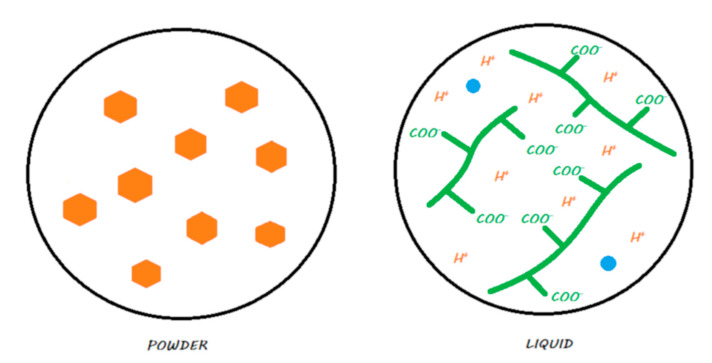
Conventional GIC in its storage medium. The powder contains FAS fillers that are not silanated, whereas the liquid contains water and ionized polyacrylic acids.

Two commercial forms exist for these materials: a manual powder–liquid mixture or a predosed capsule that needs to be mechanically vibrated. Figure 2 explains the locations of the compartments (powder or liquid) of the material.

**Figure 3:**
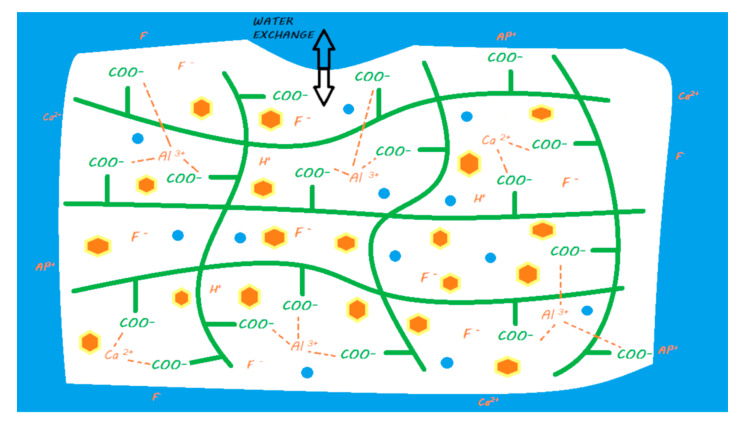
Ion release processes from a conventional GIC once it is in a moist environment (i.e., an oral environment). When the powder and liquid are mixed, the acid–base reaction is initiated, the setting of the material begins, and the FAS fillers are partially attacked. A silicic gel is partially formed on the FAS filler surface. The released calcium and aluminum ions are able to form ionic bonds with the ionized carboxylic groups. Fluoride ions are also released. In water, calcium, aluminum, and fluoride ions (and eventually other ions) are able to be exchanged with the oral environment.

During the mixing of the powder and liquid, the acid–base reaction begins and consists of an attack of the FAS fillers by the polyacrylic acid followed by an ion release according to their composition [17]. This release of calcium and aluminum ions in particular initiates a gelling reaction via ionic bonds with the ionized carboxyl ions of the polyacrylic acids. Polyacrylate salts, first of calcium and then of aluminum, are thus formed in the course of the reaction [18]. During the first few minutes of the reaction, the material remains very sensitive to a possible alteration in its water balance [19,20]. In addition, a silicic gel resulting from the acid–base reaction is formed on the surface of the partially reacted FAS fillers, allowing them to be fixed well to the matrix and protecting them from hydrolysis by considerably increasing the insolubility of the cement; this reaction is essential to the stability of the GIC [14,21] (Figure 3). This resistance to hydrolysis is very important, as this material contains between 20% and 25% water in its final structure [22].

This setting reaction is very slow. Despite a relatively short working time of a few minutes, acceptable mechanical properties need more time to be reached and are largely established after 24 h. The setting reaction continues over several weeks or even months; this is called the maturation process [14,23,24,25].

Due to their purely acid–base reaction, these materials have a true bulk-fill setting reaction.

#### 2.1.2. Mechanical Properties, Ion Release in the Oral Environment, and Indications as a Definitive Restorative Material

These conventional GICs have low flexural values [26] and a high propensity to wear [27].

The ion release of GICs in the oral environment varies depending on the composition of the FAS fillers. However, fluoride, aluminum, and calcium ions are considered to be the main ions released [17,25] (Figure 3). In the majority of studies, conventional GICs are the materials that release the highest level of fluoride among the studied materials [28]. An ion release peak is observed in the first hours of material placement [29,30,31,32,33,34]. This release tends to decrease over time until it reaches a plateau. This material is capable of recharging itself, especially with fluoride ions from the oral environment [28,34,35]. This ion release has been shown to induce remineralization of the underlying hard dental tissues in many in vitro studies [36,37]. Therefore, conventional GICs can be considered bioactive restorative materials.

Today, these conventional GICs are virtually no longer used for definitive restorations. The last indication, with a satisfactory success rate, is prophylactic fissure sealing in pediatric dentistry [38,39].

### 2.2. High-Viscosity Glass Ionomer Cements (HV-GICs)

#### 2.2.1. Composition and Chemical Reactions

Although the term HV-GIC is widely used in international publications, some authors continue to place these products in the category of conventional GICs [40,41,42]. These HV-GICs incorporate small FAS fillers in addition to others to increase the speed of the reaction. The latter benefit from a surface treatment (patented), which increases their reactivity. The powder/liquid ratio increases [43,44,45,46,47,48,49], as does the molecular weight of polyacrylic acid [50].

**Figure 4:**
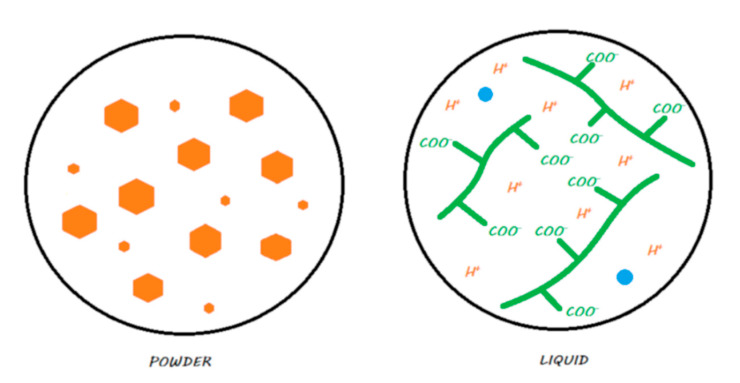
HV-GIC in its storage medium. The powder contains nonsilanated FAS fillers in which very small FAS fillers are added to speed up the reaction and increase the powder/liquid ratio. The liquid contains water and ionized polyacrylic acids.

Two commercial forms exist: manual powder–liquid mixtures or predosed capsules that need to be mechanically vibrated. Figure 4 explains the locations (powder or liquid) of the main components of the material.

**Figure 5:**
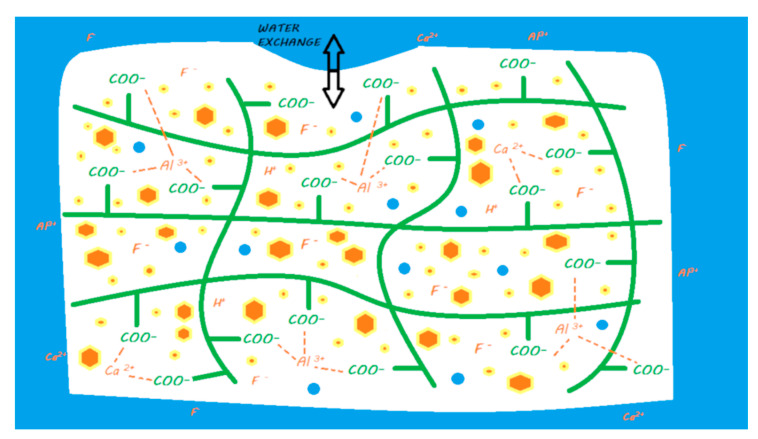
Ion release from an HV-GIC once it is in a moist environment (i.e., an oral environment). The mechanism of the reaction and ion exchange with the oral environment are the same as that previously described for conventional GIC.

When mixing the liquid powder, the steps and setting characteristics are the same as those previously described for conventional GICs (Figure 5). They also maintain a true ‘bulk-fill’ reaction. Due to chemical innovations, the initial setting time is reduced to limit the water balance sensitivity [44,49].

In addition, the use of these materials is associated with a photopolymerizable varnish that isolates them from the oral environment during the initial stages of the setting reaction, thus reducing their sensitivity to alterations in the water balance [51,52].

#### 2.2.2. Mechanical Properties, Ion Release in the Oral Environment, and Indications as a Definitive Restorative Material

All these modifications substantially increase the flexural strength of the material and thus reduce the risk of cohesive fracture of the material, which is reported to be the major cause of failure of conventional GICs [53,54,55]. Their wear resistance is now clinically acceptable [56].

The release and recharge mechanisms of HV-GICs are similar to those described for conventional GICs (Figure 5), including a release peak at placement. However, HV-GICs generally appear to release less fluoride than their precursors, conventional GICs [28,57]. This ion release has been shown to induce remineralization of the underlying hard dental tissues in many in vitro studies [18,36,58,59,60]. Therefore, HV-GICs can be considered bioactive restorative materials.

Compared with conventional GICs, these materials have wider indications for use in restoration. Thus, they can be successfully used as restorative materials for Class I and Class II limited occlusal restorations in adults [61], cervical restorations [62], in children, or as an intermediate base in the sandwich technique [63].

### 2.3. Resin-Modified Glass Ionomer Cements (RM-GICs)

#### 2.3.1. Composition and Chemical Reactions

RM-GICs retain the same acid–base reaction as GICs combined with a radical polymerization reaction of methacrylate monomers [64]. Monomers are added to the liquid (typically 2-hydroxyethyl methacrylate (HEMA)), as well as photoinitiators such as camphorquinone. The purpose of resin addition is to decrease the setting time, enhance the mechanical properties, and decrease the sensitivity of the material to early aqueous or salivary contamination compared to conventional GICs [28].

The fillers are silanated FASs [65,66,67]. This silanization treatment of reactive fillers has not been adequately communicated by manufacturers, which theoretically allows the binding of these FAS particles to the resin matrix, further increasing the cross-linking of the resin network, improving the final mechanical properties of the material and modulating the solubilization of the reactive fillers [68,69,70,71]. However, the stability of the silanization of FAS fillers and its impact on ion release, in parallel with the acid–base reaction, is unclear and has not been studied.

**Figure 6:**
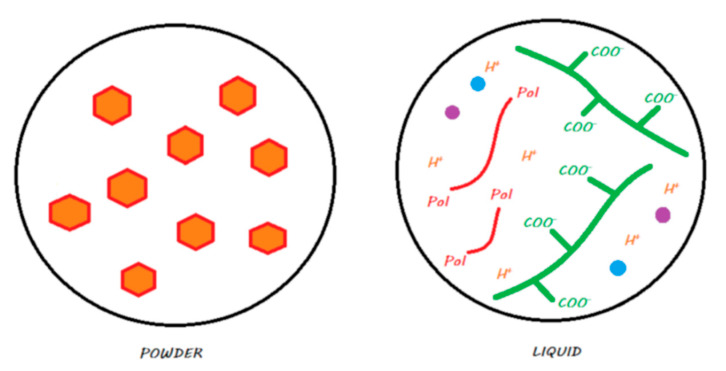
An RM-GIC in its storage medium. The powder, in contrast with GICs and HV-GICs, contains silanated FAS fillers; the liquid contains the same components as those in the GIC liquid but with HEMA monomers added to the formulation.

Two commercial forms of these materials are available: manual powder–liquid mixtures or the predosed capsules to be mechanically vibrated. Figure 6 explains the locations of the compartments (powder or liquid) of the main components of the material.

**Figure 7:**
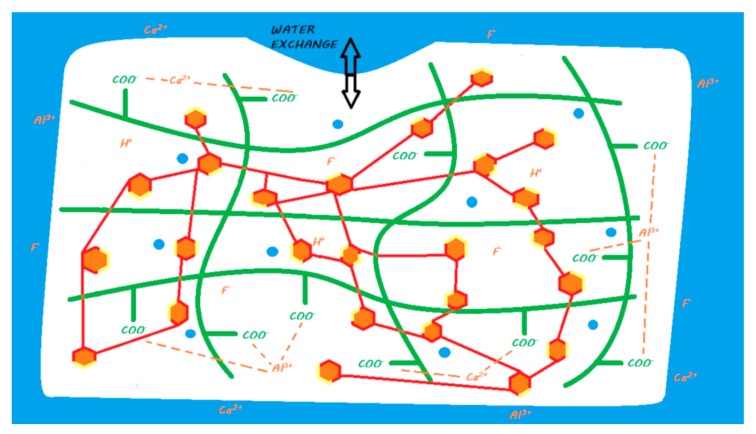
Ion release from an RM-GIC once it is in a moist environment (i.e., an oral environment). When the powder and liquid are mixed, the acid–base reaction is initiated, the setting of the material begins, and the FAS fillers are partially attacked: a silicic gel is partially formed on the FAS filler surface. The released calcium and aluminum ions are able to form ionic bonds with ionized carboxylic groups. Fluoride ions are also released. A second reaction of resin polymerization is activated when the material is light-cured: monomers can copolymerize with other monomers or silanated FAS fillers. At the end of the reaction, two different interpenetrated networks are produced without covalent or ionic links between both. In water, calcium, aluminum, and fluoride ions (and eventually other ions) are able to be exchanged with the oral environment.

When mixing a powder and liquid, two reactions occur: an acid–base reaction, similar to that of a conventional GIC, and a polymerization reaction triggered by light activation (Figure 7). The setting of this material can be described as a dual setting process. However, these materials have a radical resin polymerization reaction that is activated only by light energy; therefore, to ensure a true dual setting throughout the thickness of these materials, they must be laminated in 2 mm layers. These materials do not allow a true bulk-fill reaction due to the absence of chemical initiators in the resin polymerization process.

Even if these two reactions coexist, they compete with each other. As soon as resin polymerization is initiated, the acid–base reaction is limited [72,73,74]. The polymerization reaction is also impacted by the acid–base reaction [75], and the unreacted HEMA monomers cause greater water absorption than that of HV-GIC due to their hydrophilicity [76]. This effect increases the sensitivity of the material to hydrolysis [77].

#### 2.3.2. Mechanical Properties, Ion Release in the Oral Environment, and Indications as a Definitive Restorative Material

The mechanical properties of RM-GICs, especially in flexion, are increased compared with those of conventional GICs and HV-GICs [26,78]. However, their resistance to wear in areas of mechanical stress remains low [27].

The release and recharge mechanisms of RM-GICs are similar to those described for conventional GICs, in particular, exhibiting a release peak when they are installed (Figure 7). However, RM-GICs appear to release less fluoride than resin-free GICs, conventional GICs, and HV-GICs [28,57,79]. The polymerized resin matrix limits ion exchange with the external environment [57]. This ion release has been shown to induce remineralization of underlying hard dental tissues in many in vitro studies [37,80,81,82]. Therefore, former RM-GICs can be considered bioactive restorative materials.

Given the limitations mentioned above and the advantages of HV-GICs, their indications are becoming increasingly limited [83]. However, they can still be used with good success rates as an intermediate base in the sandwich technique [84], for the restoration of cervical lesions [62], for primary teeth under certain conditions, or when the operator wishes to have a controlled set of the material, which is not possible with conventional GICs and HV-GICs.

### 2.4. Special Cases of Vitremer and Its Evolution: Ketac Nano (Also Called Ketac N100)

#### 2.4.1. Composition and Chemical Reactions

To describe the chemistry of these materials, many international studies evaluated the behavior of Vitremer (3M ESPE, St. Paul, MN, USA). Vitremer appeared in 1991, one year after the first RM-GIC; it was immediately categorized as a member of this family despite its chemical characteristics [15] due to its similar behavior in terms of mechanical properties and fluoride release [24,55,85,86,87,88,89]. Ketac Nano (3M ESPE, St. Paul, MN, USA) is an evolution of Vitremer that was introduced in 2007. We chose to specifically describe the chemistry of these materials because they represent major evolutions in the chemistry of RM-GICs, which will serve as elements to justify our classification and to understand the chemistry of the new commercial products.

The Vitremer matrix is based primarily on the use of functionalized high molecular weight polyacrylic acids. Schematically, they are the same polyacrylic acids as those found in the matrix of HV-GICs and RM-GICs, but some –COOH groups are substituted by methacrylate groups with a carbon–carbon double bond, allowing a polymerization reaction (called Vitrebond copolymer by the manufacturer). This is the first major innovation of this product, as it theoretically allows improved cross-linking between resinous and polyacid networks, although no studies have been conducted to investigate this particular issue. The product also contains other acids frequently found in GICs, HEMA monomers identical to those in other RM-GICs and water to activate the acid–base reaction. Finally, it contains chemopolymerization agents in addition to photopolymerization agents. This chemopolymerization ensures a theoretical deep resin setting between the methacrylate groups of the functionalized polyacrylic acids. This development is an advance compared to the classic RM-GICs; therefore, it is a theoretical bulk-fill reaction [90]. However, studies on the depth of polymerization of Vitremer, compared with other RM-GICs without chemopolymerization activators, have not reported any obvious differences and still recommend a layering technique [85,91].

The fillers are silanated FAS.

Despite the innovations described above, the behavior of the material in the short and long term remains similar to that of the RM-GICs that do not benefit from these innovations or whose trade secrets have not been disclosed.

**Figure 8:**
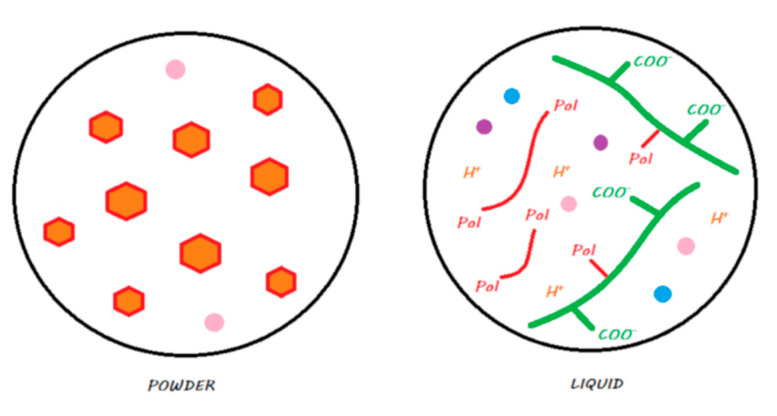
Vitremer in its storage medium. The powder contains silanated FAS fillers, and some chemopolymerization components are added. The liquid contains water, an ionized modified polyacrylic acid with photopolymerizable groups, camphorquinone as a photoinitiator, and some chemopolymerization components.

This material is in the form of a powder–liquid mixture that is spatulated manually. Figure 8 explains in which compartments (powder or liquid) the main components of the material are located.

**Figure 9:**
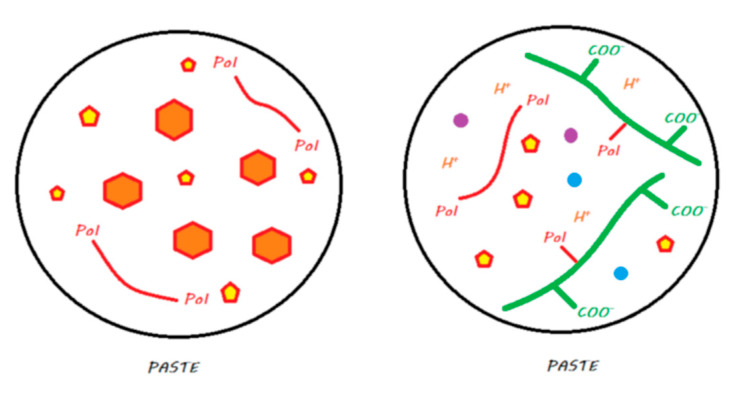
Ketac Nano in its storage medium. The first paste contains silane-treated reactive FAS fillers and unreactive fillers, as well as HEMA monomers. The second paste contains water, an ionized modified polyacrylic acid with photopolymerizable groups, camphorquinone as a photoinitiator, a blend of monomers including HEMA, and silane-treated unreactive fillers.

Ketac Nano, which was introduced in 2007 [15], is the chemical evolution of Vitremer and is provided in the form of a single-use self-mixing capsule. It represents an evolution in terms of manipulation. This material always requires the application of a primer on the tooth surface before being inserted into the cavity and does not have the chemopolymerization activators contained in Vitremer; it must therefore be laminated in 2 mm layers as it has no bulk-fill properties [92]. The defining feature of this material, in addition to possessing a more varied monomeric composition, is that it contains silanized nanofillers and nanoclusters. The idea of this modification is to improve its mechanical properties, as shown in the literature on experimental RM-GICs [93]. This concept, as shown below, is also used for Activa BioActive Restorative and Surefil One. Figure 9 explains the compartments (paste/paste) in which the main components of the material are located.

**Figure 10:**
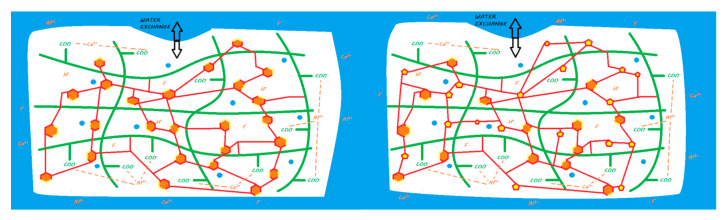
Ion release from Vitremer (left) or Ketac Nano (right) once they are in contact with a moist environment (i.e., oral environment). When the powder and liquid (or pastes for Ketac Nano) are mixed, the acid–base reaction is initiated, the setting of the material begins. Once the FAS fillers are partially attacked, a silicic gel partially forms on the FAS filler surface. The released calcium and aluminum ions are able to form ionic bonds with ionized carboxylic groups. Fluoride ions are also released. A second reaction involving resin polymerization occurs during mixing for Vitremer (chemopolymerization) or during light curing for Ketac Nano. Monomers can copolymerize with silanated FAS fillers and other monomers for both materials and silanated unreactive fillers for Ketac Nano. At the end of the reaction, two interconnected networks are obtained with covalent links between both due to the modified polyacrylic acid (Vitrebond copolymer). In water, calcium, aluminum, and fluoride ions (and eventually other ions) are able to be exchanged with the oral environment.

Upon activation of the mixture, the setting reaction of these two materials is similar to that of RM-GICs (Figure 10). Because of the attack of the carboxylic acids on the functionalized polyacid, ion salting-out occurs due to the silanized FAS fillers, and a layer of silicic gel partially forms on the surface. The parts of the reactive filler that remain silanized enable its linkage to the resin network.

For Ketac Nano, an additional bond between the resin matrix and the silanized nanofillers/nanoclusters can be expected to improve its mechanical and optical properties.

The setting kinetics of Vitremer and Ketac Nano are comparable to those of other RM-GICs [74]. Competition between resinous and acid–base reactions is suspected for both materials but has not yet been studied.

#### 2.4.2. Mechanical Properties, Ion Release in the Oral Environment, and Indications as a Definitive Restorative Material

Vitremer and Ketac Nano have a fluoride release profile close to that of RM-GICs [30,88] (Figure 10), and the release peak at the time of their application is preserved. This ion release was demonstrated in many in vitro studies with Vitremer to induce remineralization of the underlying hard dental tissues [94]. No study has focused on the potential of Ketac Nano to induce remineralization. Therefore, Vitremer can be considered a bioactive restorative material. Even if Ketac Nano properties are close to those of Vitremer, information is lacking about its potential bioactivity.

The indications of Vitremer and Ketac Nano are identical according to the manufacturer and are just as limited as those of the RM-GIC. These materials can reasonably be used in the sandwich technique for the restoration of cervical lesions [95,96] or for primary teeth. Whereas Vitremer is a material with a long clinical record of accomplishment, there are few clinical studies for Ketac Nano [97,98,99].

#### 2.4.3. Update on the Classification

Because of the chemistry of Vitremer, it seems consistent to classify it with RM-GICs. It has already been extensively studied in the literature, and its performance is consistent with that of the other products in this family. Its chemistry is not fundamentally different from that of RM-GICs since it is based on reactive FAS fillers and a polyacrylic acid, albeit a functionalized polyacrylic acid. The addition of chemoinitiators and silanization of FAS fillers appear to have only produced a marginal effect.

Ketac Nano is sometimes classified as a nanoionomer because of the terminology provided by the manufacturer and because the nanoparticles and nanoclusters it contains are analogous to nanohybrid composites [100]. Because its chemistry is close to that of Vitremer and as the strategy of adding silanized nonreactive particles has already been proposed to create reinforced RM-GICs [93], it seems reasonable and consistent to classify it in the category of RM-GICs, especially because the increase in its clinical indications is limited and few studies classifying it as a nanoionomer have been published.

## 3. Fluoride-Releasing Composites

### 3.1. Compomers

The term ‘compomer’ is a combination of ‘composite’ and ‘ionomer’. In addition to this generic term, these materials are also called polyacid-modified composite resins [6]. After a long debate on terminology [101], the term compomer finally became a part of scientific language [102].

#### 3.1.1. Composition and Chemical Reactions

These compomers have two major differences with resin composites: one involves organic monomers and the other involves inorganic fillers [102].

The organic resin component in compomers is similar to that possibly contained in a composite, with a base around bisphenol A-glycidyl methacrylate (bis-GMA) and other monomers added to modify its rheological and polymerization properties (such as triethylene glycol dimethacrylate (TEGDMA) and urethane dimethacrylate (UDMA)) [103]. As the vast majority of these formulations are photopolymerizable, they also contain photoinitiators, such as camphorquinone [104]. In addition, a small proportion of functional monomers with carboxylic acid (–COOH) groups is added to the chemical composition, which indicates the first major characteristic of compomers, hence the name ‘polyacid-modified composite resins’. As this material contains no trace of water in its composition, the acid groups are dehydrated in the –COOH form, and once the material has polymerized, it is perfectly incorporated into the resin matrix [103]. Despite the addition of dehydrated acid, the final material obtained is highly hydrophobic.

The second unique feature of compomers regarding the inorganic mineral component is the presence of silanated reactive FAS fillers [105,106] in addition to silanated nonreactive quartz or silica fillers, which constitute the major part of the matrix; these reactive fillers can bind to the resin matrix, improving the mechanical properties, and releasing fluoride ions under certain conditions.

**Figure 11:**
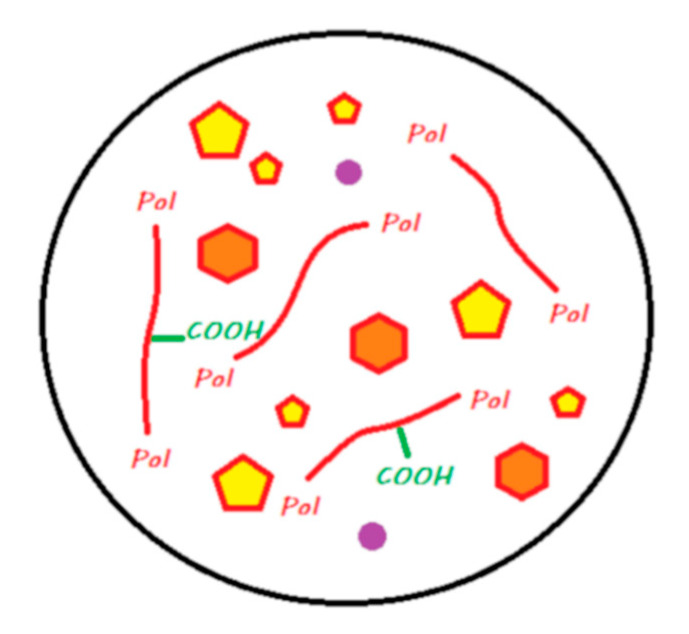
A compomer in its storage medium. This material contains, schematically, silane-treated reactive FAS fillers and unreactive fillers, a blend of monomers including dehydrated acidic monomers and camphorquinone, but does not contain any water.

These materials come in the form of single-use compules or reusable tubes. Figure 11 shows the main components of a compomer in its storage medium before polymerization.

The setting reaction of these materials is initiated by photopolymerization, where the energy supplied causes the activation of photoinitiators and results in radical polymerization that is similar to that of a resin composite [45]. A resin network is formed, and covalent bonds are formed with silanated FAS and silanated nonreactive fillers (Figure 11). Compomers do not exist at the moment with bulk-fill properties, and they have to be laminated in 2 mm layers.

#### 3.1.2. Mechanical Properties, Ion Release in the Oral Environment, and Indications as a Restorative Material in Use

Compomers have initial mechanical properties that are roughly comparable to those of resin composites [106,107,108], but their performance decreases significantly over time due to hydrolysis and solubilization of the fillers [106,109,110]. The stability of the silanization of FAS fillers in parallel with the acid–base reaction of FAS feeds is, as with Vitremer and Ketac Nano, subject to questions in the event of hydrolysis [106] and has not been studied.

**Figure 12:**
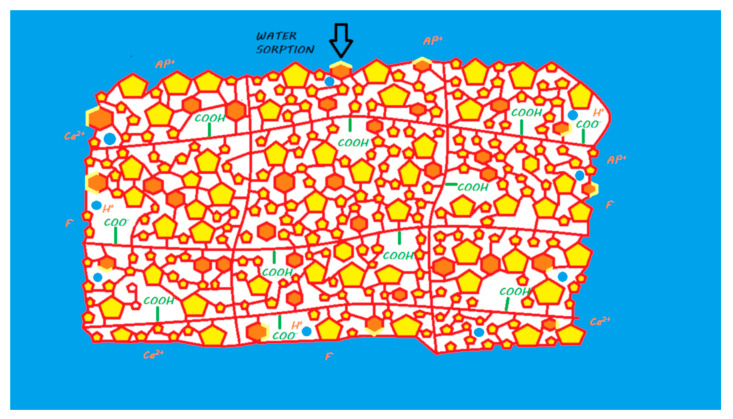
Ion release from a compomer once it is in contact with the oral environment. When the material is light cured, a resin polymerization reaction is initiated, and monomers can copolymerize with other monomers, silanated FAS fillers, and unreactive fillers. The acidic groups remain dehydrated, and no acid–base reaction occurs in the setting reaction of the material. When placed in a moist environment (i.e., oral environment), water sorption occurs, and dehydrated acidic monomers located in the periphery of the material can release protons to attack silanated FAS fillers. This mechanism leads to the release of calcium, aluminum, and fluoride ions. These ions do not participate in the setting mechanism of the material.

Compomers with no water in their composition exhibit ion release based solely on the absorption of water that occurs after contact with the oral environment [45]. Compared with that of a resin composite, this water absorption is facilitated by silanated FAS fillers, which increase water absorption compared with that of silanated nonreactive fillers [105]. This process occurs at the periphery of the material and, when it comes into contact with a dehydrated acid, causes its activation in its ionized form (COO– + H+); the proton that is released is able to attack the surface of the FAS fillers and induce ion release; in particular, fluoride is released [101] (Figure 12).

The ion release of these materials is very low and much lower than that of conventional GICs, HV-GICs, and RM-GICs. In addition, the initial peak in the fluoride release observed for conventional GICs, HV-GICs, and RM-GICs is no longer observed for these materials, which are still capable of recharging with fluoride [31,79,111,112,113,114]. Compomers appear to have an ion release close to that of giomers [31,79]. This small ion release has not been shown to induce remineralization of underlying hard dental tissues in vitro [115]. Compomers require an adhesive to be bonded on the tooth structure, which could impede ion diffusion to induce remineralization. Therefore, compomers cannot be considered bioactive restorative materials.

Although they appear to have a lower long-term success rate than that of composite resins in some studies [116], compomers can be reasonably used long term for cervical restorations [95,117,118], anterior restorations [119,120], as an intermediate base under adult posterior composite restorations [121,122], or for restorations in pediatric dentistry [121,123,124].

#### 3.1.3. Update on the Classification

As their setting chemistry is comparable to that of resin composites (absence of water, setting by resin polymerization, and silanized fillers), where they differ only by a mechanism of postpolymerization ion release by water absorption, we propose to classify compomers in a family called ion-releasing composites (IRCs).

### 3.2. Giomers

The term ‘giomer’ is an English combination of ‘glass ionomer cement’ and ‘resin composite’. Unlike compomers that incorporate lyophilized modified resin groups and initially inactivated reactive fillers, giomers use dehydrated and silanized preactivated reactive fillers [125,126].

#### 3.2.1. Composition and Chemical Reactions

The resin matrix in a giomer is similar to that possibly contained in the composite, with a base around bis-GMA and other monomers added to modify its rheological and polymerization properties [127]. No functional acid groups or dehydrated acid groups are incorporated in the composition; the material therefore lacks adhesive potential and requires the use of an adhesive. Setting is performed by photopolymerization, and the photoinitiators found are similar to those contained in resin composites [128]. The main feature of a giomer is that in addition to the nonreactive silanized glass fillers of the resin composite, it incorporates reactive fillers of preactivated FAS (i.e., coated with SiO_2_ gel), similar to those contained in HV-GICs after the setting reaction [127]. For this purpose, before their incorporation into a material that already contains organic monomers and silanized nonreactive glass fillers, the FAS fillers are pre-etched with polyacrylic acid to cover them with a silicic gel, dehydrated by freeze-drying, and functionalized by silanization to allow their copolymerization with the resin monomers and to make them suitable for ion release in contact with water when it is absorbed into the material [129].

The fillers obtained at the end of this treatment are called pre-reactive glass ionomer particles (PRGs). According to the volume of activation obtained from the FAS filler at the end of the treatment, a distinction is possible between S-PRG fillers (only the surface of the filler is activated by the chemical treatment) and F-PRG fillers (the entire filler or almost the entire filler has been activated by the chemical treatment) [129]. For restoration giomers, S-PRG technology is used to treat reactive fillers.

**Figure 13:**
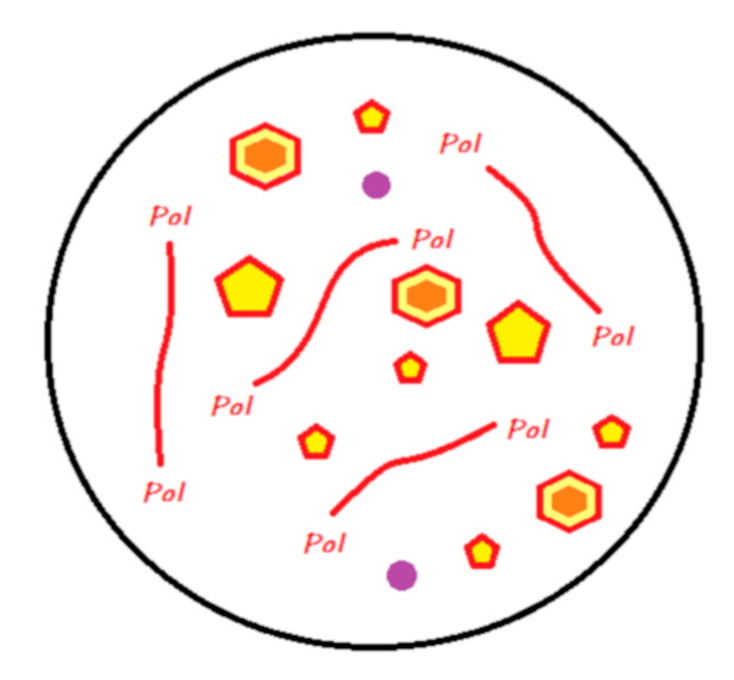
A giomer in its storage medium. This material contains, schematically, a silane-treated partially pre-reacted FAS filler (S-PRG), an unreactive filler, and a blend of monomers and camphorquinone. It does not contain any water.

These materials, marketed by the Shofu Company, are provided in the form of single-use compules or reusable tubes. Figure 13 shows the main components of a giomer in its storage medium before polymerization.

**Figure 14:**
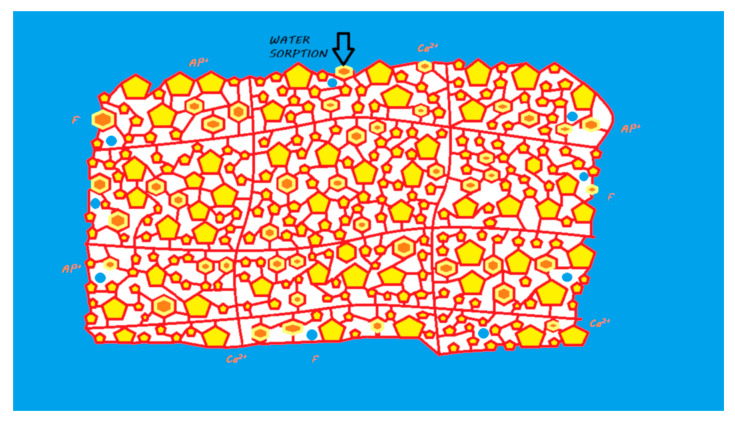
Diagram of ion release from a giomer once it is in contact with the oral environment. When the material is light cured, a resin polymerization reaction is initiated, and monomers can copolymerize with other monomers, silanated S-PRG fillers, and unreactive fillers. No acid–base reaction occurs in the setting reaction of the material. When placed in a moist environment (i.e., oral environment), water sorption occurs, and S-PRG fillers are able to release calcium, aluminum, and fluoride ions. These ions do not participate in the setting mechanism of the material.

The setting reaction of a giomer occurs by photopolymerization; this process is similar to that in a composite or compomer that involves the creation of a resin network and the establishment of covalent bonds with silanized reactive S-PRG and silanized nonreactive fillers (Figure 14). Giomers exist in laminable or bulk-fill versions.

#### 3.2.2. Mechanical Properties, Ion Release in the Oral Environment, and Indications as a Restorative Material in Use

Studies on the initial properties of giomers reported initial mechanical properties that are comparable with those of a resin composite [130,131].

As with compomers, the ion release of this material is based on the absorption of water that occurs once it comes into contact with the oral environment [127,132]. This water absorption occurs at the periphery of the material, and when it comes into contact with an S-PRG filler, it causes ion release; this S-PRG filler must be viewed as more reactive than a conventional FAS filler. Therefore, there is no need for an acid to cause the activation of the reactive filler, unlike for compomers (Figure 14).

However, the ion release of these materials is shown to be very low and much lower than that of HV-GICs and RM-GICs. It is comparable to that of compomers, and as with compomers, no fluoride release peak is observed after the placement of giomers [31,79,111,112]. However, giomers are capable of being recharged with fluoride ions [133]. This small ion release ability that induces remineralization of the underlying hard dental tissues has not been studied. However, as with compomers, giomers require an adhesive to be bonded on the tooth structure, which could impede ion diffusion to induce remineralization. Starting from this base, giomers cannot be considered bioactive materials.

Although they appear to have a lower long-term success rate than resin composites in some studies [128], giomers can be reasonably used for cervical restorations [134], occlusal restorations [134], or restorations in pediatric dentistry. Reservations were expressed by some authors on the esthetic rendering of giomers in the long term [135].

#### 3.2.3. Update on the Classification

Given that the setting chemistry of giomers is comparable to that of resin composites (an absence of water, setting by resin polymerization, and silanized fillers), and they differ only by a mechanism of postpolymerization ion release by water absorption, we propose to classify them in the family of ion-releasing composites (IRCs).

These giomers are ultimately similar to compomers. S-PRG technology is used to produce additional reactive silanized FAS particles to avoid the use of a dehydrated acid, which is present in compomers.

## 4. New Bioactive Composites

These materials were isolated from PubMed studies on fluoride-releasing materials. The names and compositions of these materials are given in Table 1. They have all been recently introduced and have therefore received little or no attention in the scientific literature. 

### 4.1. Activa BioActive Restorative

To describe the chemistry of this material that was introduced in 2013, we have a few independent sources of scientific publications and information from the patent for this product [136]. Activa BioActive Restorative is referred to by its manufacturer and some authors as a “bioactive composite” [137], but it is considered by others to be an RM-GIC [138,139].

#### 4.1.1. Composition and Chemical Reactions

The Activa BioActive Restorative liquid contains a high molecular weight polyacrylic acid similar to that used in HV-GICs and RM-GICs and not modified by methacrylate polymerizable groups, as in Vitremer or Ketac Nano. Urethane dimethacrylate monomers (called Embrace resin by the manufacturer) and dimethacrylate phosphate (acids) are added. To initiate the polymerization reaction, the material contains photoinitiators and chemical initiators. Finally, this liquid contains a small proportion of water.

The fillers are silanized FAS fillers and silanized nonreactive fillers that are able to bond with the resin matrix and play a role in the wear resistance and esthetics of the material.

**Figure 15:**
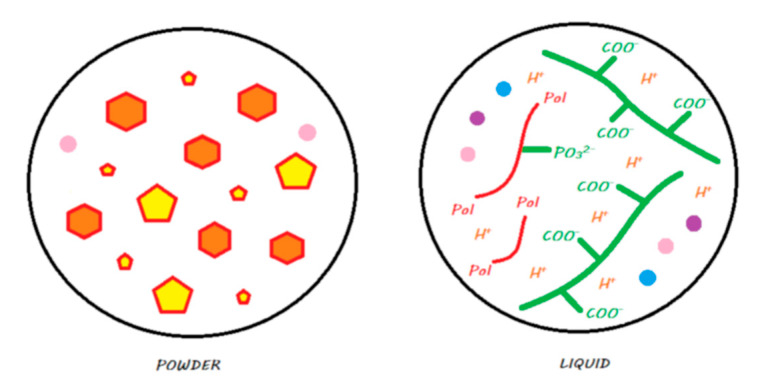
Diagram of Activa BioActive Restorative in its storage medium. The powder contains silanated FAS fillers, some chemopolymerization components, and silanated unreactive fillers. The liquid contains water, polyacrylic acid, a blend of monomers including phosphate dimethacrylate monomers, camphorquinone as a photoinitiator, and some chemopolymerization components.

This material is in the form of a self-mixing syringe and is theoretically a true bulk-fill material. Figure 15 explains the compartments (powder/liquid) in which the main components of the material are located.

A double-setting reaction occurs during mixing. An acid–base reaction occurs, with polyacrylic acid and dimethacrylate phosphate monomers attacking silanized FAS fillers. This acid–base reaction causes an ion release similar to that described for previous materials. Along with this acid–base reaction, a resin polymerization reaction is activated upon mixing by the chemopolymerization activators and then completed by photopolymerization. The silanized FAS fillers are theoretically capable of binding to the resin matrix, as are silanized nonreactive fillers.

**Figure 16:**
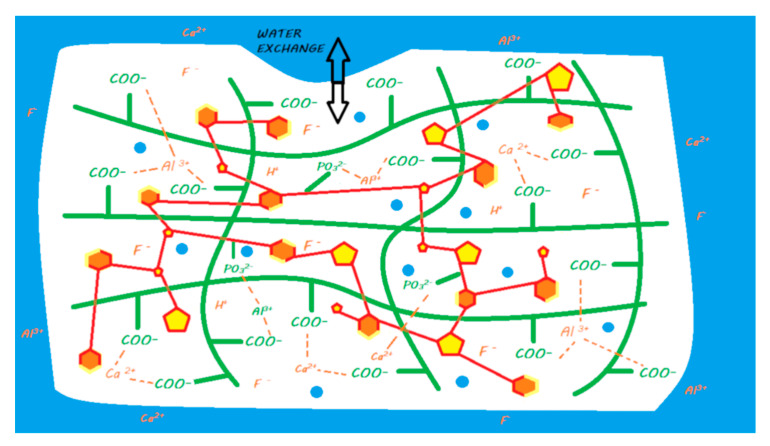
Diagram of ion release from Activa BioActive Restorative once it is in contact with a moist environment (i.e., oral environment). When the powder and liquid are mixed, the acid–base reaction is initiated, the setting of the material begins, and the FAS fillers are partially attacked. A silicic gel partially forms on the FAS filler surface. The released calcium and aluminum ions are able to form ionic bonds with ionized carboxylic groups. Fluoride ions are also released. A second reaction of resin polymerization occurs during mixing: monomers can copolymerize with silanated FAS fillers, silanated unreactive fillers, and other monomers. At the end of the reaction, we obtain two different interpenetrating networks with theoretical ionic links between both with trivalent ions. In water, calcium, aluminum, and fluoride ions (and eventually other ions) are able to be exchanged with the oral environment.

The ions released by the acid–base reaction is either exchanged with the external environment or creates ionic bonds between polyacrylic acids, but trivalent ions, such as aluminum, also bind to polyacrylic acid and dimethacrylate phosphate, thus cross-linking the resin and ionic networks (Figure 16).

The same mechanisms as those described for RM-GICs regarding competition between the acid–base and resin polymerization reactions are suspected to be present in this material.

#### 4.1.2. Mechanical Properties, Ion Release in the Oral Environment, and Indications as a Restorative Material for Use

Regarding the initial evaluation of the mechanical properties of Activa BioActive Restorative, its in vitro wear was shown to be comparable to that of a hybrid composite and inferior to that of RM-GICs and HV-GICs [137,140]. Its initial flexural strength is superior to those of RM-GICs and HV-GICs. The results for composites and their derivatives (compomers and giomers) are contradictory but appear to be similar [137,138,141].

The fluoride release of Activa BioActive Restorative appears to be lower than that of RM-GICs and HV-GICs [142,143] and close to that of compomers and giomers, both quantitatively and qualitatively; it lacks an initial release peak but possesses the capacity to be recharged by fluoride [137,142]. In addition to the release of calcium and fluoride, the release of phosphate is noted by the manufacturer. This ion release has not been found to induce remineralization of underlying hard dental tissues in vitro. Therefore, Activa BioActive Restorative cannot be classified as a bioactive material at the moment [144].

Due to its recent introduction, no long-term clinical studies are available on this material. The manufacturer specifies this material for all direct anterior and posterior restorations. It is also indicated as an intermediate base.

Two short-term clinical studies report different behaviors for posterior restorations for use in adults. In the absence of an adhesive (possibly an old protocol that is not currently recommended by the manufacturer), the short-term results obtained with Activa BioActive Restorative are contradictory. One study reported good short-term behavior, and the other reported an unacceptable failure rate [139,145]. The new protocol proposed by the manufacturer involves the use of an adhesive system prior to the application of Activa BioActive Restorative.

#### 4.1.3. Update on the Classification

This material is therefore similar to RM-GICs, exhibiting a double-setting reaction and presenting similarities to Vitremer (setting of a dual resin matrix and silanization of FAS fillers) and Ketac Nano (presence of silanized nonreactive fillers). It also has the distinction of using a dimethacrylate phosphate monomer that, once ionized, is theoretically able to cross-link resin and acidic networks through aluminum cations.

Despite these innovations, Activa BioActive Restorative could be classified as an RM-GIC. It cannot be considered a resin composite; these composites and their derivatives do not contain water and are not derived, for the most part, from an acid–base reaction. However, this material is quite different from the first generation of RM-GICs due to its reinforced formulation.

### 4.2. Cention N

To describe the chemistry of this material, we have a few independent sources as well as the product patent [146]. Please note that unlike the other products described in this article, Cention N is currently only marketed in Asia. The manufacturer places it in a new family that was apparently derived from the family of composites called alkasites. This name is used in the first international publications on this product.

#### 4.2.1. Composition and Chemical Reactions

The liquid of Cention N is composed of the association of four monomers commonly found in the composition of resin composites. It does not contain any acidic monomer or water; the material is therefore devoid, a priori, of adhesive potential (the manufacturer indicates this with an adhesive in nonretentive cavities). The liquid also contains photopolymerization and chemopolymerization activators and is therefore, theoretically, a true bulk-fill material.

The defining feature of this material lies in the composition of its powder, particularly in the reactive fillers that it incorporates. In addition to nonreactive silanized fillers, Cention N has reactive silanized FAS fillers similar to those used in GICs (calcium-barium-aluminum-fluorosilicate-glass) and silanized fillers advertised as highly reactive particularly in an acidic environment, which strongly resembles FAS [144] (calcium fluorosilicate glass) fillers. These fillers are the origin of the name ‘alkasite’ given by the manufacturer.

**Figure 17:**
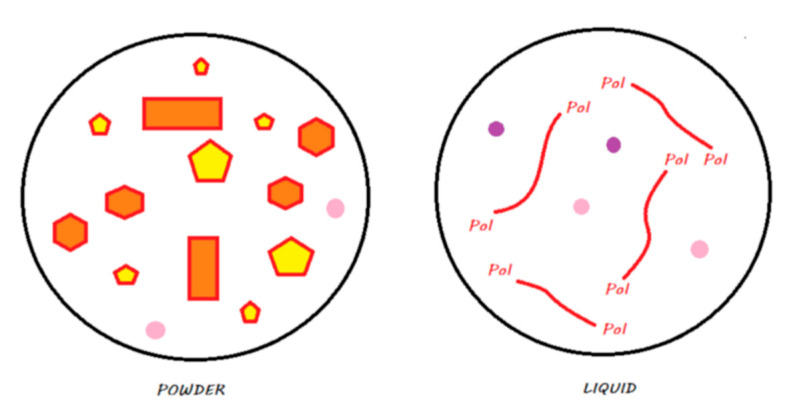
Diagram of Cention N in its storage medium. The powder contains, schematically, silane-treated FAS filler, silanated ‘alkasite’ filler, unreactive filler, and some chemopolymerization components. The liquid contains a blend of monomers, Ivocerin as a photoinitiator, and some chemopolymerization components. It does not contain any water.

This material is in the form of a powder–liquid mixture and must be spatulated manually. Figure 17 explains compartments (powder/liquid) in which the main components of the material are located.

**Figure 18:**
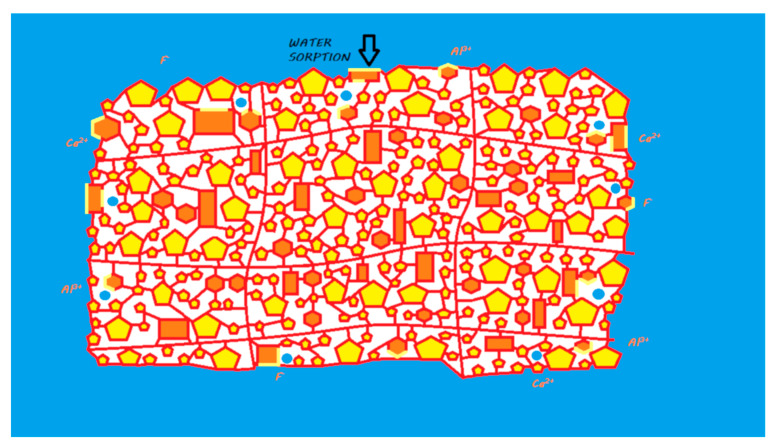
Diagram of ion release from Cention N once it is in contact with the oral environment. When the material is mixed, a resin polymerization reaction is initiated due to the chemical initiator, and monomers can copolymerize with other monomers, silanated FAS filler, silanated alkasite fillers, and unreactive fillers. No acid–base reaction occurs in the setting reaction of the material. When placed in a moist environment (i.e., oral environment), water sorption occurs in FAS fillers, and alkasite fillers are able to release calcium, aluminum, and fluoride ions (and eventually other ions). These ions do not participate in the setting mechanism of the material.

The radical polymerization reaction starts with a chemopolymerization reaction as soon as the liquid–powder mixture is mixed and is completed by photopolymerization. This setting reaction is similar to that of a composite, giomer, or compomer, with the creation of a resin network and the establishment of covalent bonds with reactive and nonreactive silanized fillers (Figure 18).

#### 4.2.2. Mechanical Properties, Ion Release, and Clinical Performance

Once placed in the oral environment, especially in an acidic environment [144], Cention N releases ions, especially fluoride, due to the absorption of water, as with giomers or compomers (Figure 18). The ion release of Cention N was shown to be superior to that of Activa BioActive. An in vitro study reported that Cention N is capable of forming apatite on its surface and thus remineralizing the underlying dentin when used without the application of a dental adhesive [144]. This material could therefore be considered bioactive and would thus be the first derivative of a composite resin with a proven bioactivity.

This material is indicated by the manufacturer for all temporary tooth restorations, for occlusal and proximal restorations of posterior teeth, and for cervical restorations in combination with an adhesive. However, no long- or short-term clinical studies have examined its performance due to its relatively short time in the market.

#### 4.2.3. Update on the Classification

This material, by its composition, is therefore very similar to resin composites, such as compomers and giomers. It is even more similar to the latter, as each of these materials is based on reactive fillers that do not require acids for their activation (S-PRG FAS fillers for giomers and calcium fluorosilicate fillers for Cention N). However, this material is capable of producing remineralization of the underlying hard tissue with which it is in contact. To date, this is the only commercially available ion-releasing composite that has been independently and scientifically proven to induce remineralization of the underlying dentin. We therefore propose classifying it in the family of bioactive composites. However, reservations are expressed about the bioactivity of this material when an adhesive is used before the placement of this material.

### 4.3. Surefil One

To describe the chemistry of this material, the only available independent source is its patent [147]. No independent studies have yet been published. The manufacturer classifies this material in the family of self-adhesive hybrid composites.

#### 4.3.1. Composition and Chemical Reactions

The Surefil One liquid consists primarily of a high molecular weight polyacrylic acid functionalized with polymerizable groups (called MOPOS by the manufacturer). Schematically, this polyacrylic acid resembles the Vitrebond copolymer found in Vitremer and Ketac Nano. Additionally, monomers are found in the liquid with two photopolymerizable ends (called BADEP by the manufacturer), which are added to act as a cross-linker between functionalized polyacrylic acid chains. Finally, photopolymerization and chemopolymerization agents and a certain proportion of water are present in the composition.

The fillers are silanized FAS fillers and silanized nonreactive fillers (sub- and supramicron in size) that bind with the resin matrix and play a role in the wear resistance and esthetics of the material. This material is therefore reminiscent of Ketac Nano, with a dual grip and a different load size distribution.

**Figure 19:**
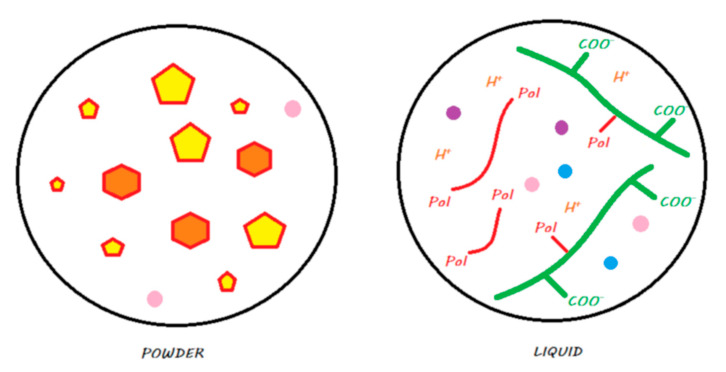
Surefil One in its storage medium. For RM-GICs, the powder contains silanated FAS fillers, some chemopolymerization components, and silanated unreactive fillers. The liquid contains water, an ionized modified polyacrylic acid with photopolymerizable groups, a blend of monomers, camphorquinone as a photoinitiator, and some chemopolymerization components.

This material is provided in the form of a single-use capsule that must be mechanically vibrated and is theoretically a true bulk-fill material. Figure 19 explains the compartments (powder/liquid) in which the main components of the material are located.

**Figure 20:**
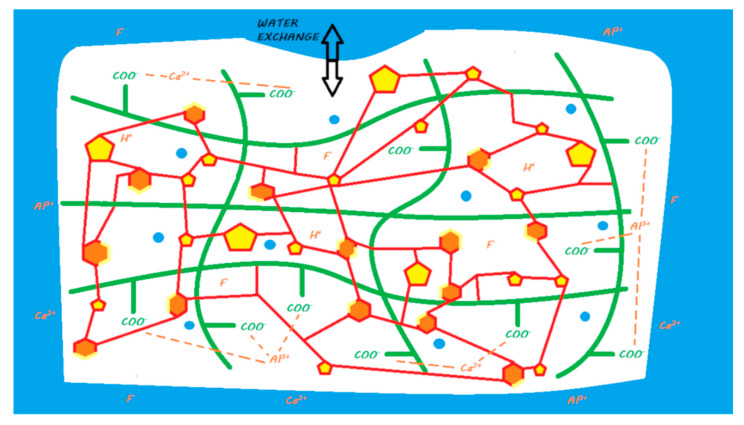
Ion release from Surefil One once in contact with a moist environment (i.e., oral environment). When the powder and liquid are mixed, the acid–base reaction is initiated, the setting of the material begins, and the FAS fillers are partially attacked. A silicic gel is partially formed on the FAS filler surface. The released calcium and aluminum ions are able to form ionic bonds with ionized carboxylic groups. Fluoride ions are also released. A second reaction of resin polymerization occurs during mixing, where monomers can copolymerize with silanated FAS fillers, silanated unreactive fillers, and other monomers. At the end of the reaction, two interconnected networks are obtained with covalent links between both due to the modified polyacrylic acid (MOPOS). In water, calcium, aluminum, and fluoride ions (and eventually other ions) are able to be exchanged with the oral environment.

During mixing, the same mechanisms as those described for Ketac Nano occur, including an attack of the silanized FAS filler by the functionalized polyacrylic acid and the subsequent ion salting-out. These released ions are either exchanged with the external environment or create ionic bonds between the functionalized polyacids. Along with the acid–base reaction, the resin polymerization reaction is initiated by the chemopolymerization activators as soon as it is mixed; it is completed by the photopolymerization reaction (Figure 20).

The same competitive mechanisms described for RM-GICs between acid–base and resin polymerization reactions are suspected in this material.

#### 4.3.2. Mechanical Properties, Ion Release in the Oral Environment, and Indications as a Restorative Material for Use

The special feature of Surefil One is that it is indicated by the manufacturer for use in all types of restorations. In vitro and clinical studies are required to assess the accuracy of these indications.

This material, which consists partly of water, theoretically promotes water and ion exchange with the oral environment. In particular, this effect leads to the release of fluoride, aluminum, and calcium ions (and probably other ions due to the composition of the reactive fillers) (Figure 20). This ion release has not yet been studied, especially its ability to induce remineralization of the underlying hard dental tissues. Therefore, Surefil One cannot be classified as a bioactive material for the time being.

To date, there are no long-term clinical studies that can be used to determine the performance of these materials.

#### 4.3.3. Update on the Classification

This material, aside from the distribution of the silanized nonreactive fillers, is very similar chemically to Ketac Nano. In addition, like Activa BioActive Restorative and Ketac Nano, this material contains water, and a significant portion of its setting is based on an acid–base reaction. As such, Surefil One represents a new evolution of RM-GICs, although it appears to have broader indications than the first generations. In the patent, the manufacturer acknowledges that the material is closely related to the GIC family [147].

## 5. Conclusions

The new fluoride-releasing materials represent a chemical evolution of adhesive–resin composite couples, compomers, giomers, and the different families of GICs used for years. These innovations correspond to something more than a simple marketing advertisement.

**Figure 21:**
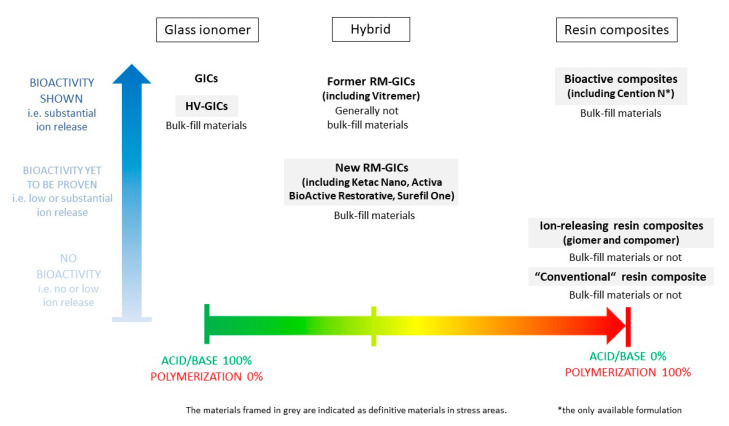
Classification of the fluoride-releasing materials according to: the importance of the acid/base reaction or resin polymerization in their setting, their bioactivity, their bulk-fill properties, and their bioactivity.

However, although the reactions of these materials are becoming increasingly hybridized among the different families described and increasingly chemically complex, we see many similarities between some of them and those of RM-GICs (for Surefil One, Activa BioActive Restorative, and Ketac Nano). These new materials represent modern evolutions of older material families and should continue to be classified among these families. Whereas the former RM-GIC was shown to be bioactive, the conservation of the bioactive properties of these new formulations has to be proven. Compomers and giomers differ from resin composites only by a mechanism of postpolymerization ion release by water absorption; we suggest classifying them in a unique family called ion-releasing composites (IRCs). The Cention N chemistry is close to that of the IRCs; this material is actually the only material that has been proven to be a real bioactive composite when used without a dental adhesive and should be classified in a new family (Figure 21).

Over time, these new formulations may tend to converge toward a composition that will provide the best time/efficiency/activity ratio. Given the lack of clinical experience with these materials, caution should be exercised in their systematic use in all patients. 

This paper provides a comprehensive overview including new materials: further work is needed to definitively validate some of the chemistries explained here, as well as to determine even more precisely the bioactivity of these materials.

## Figures and Tables

**Table 1:** Composition of new fluoride-releasing materials

Name	Composition
Activa BioActive Restorative	Powder: silanated bioactive glass and calcium, silanated silica, and sodium fluoride Liquid: diurethane modified by the insertion of a hydrogenated polybutadiene and other methacrylate monomers, modified polyacrylic acid, and water
Cention N	Powder: barium aluminum silicate glass, ytterbium trifluoride, isofiller, calcium barium aluminum fluorosilicate glass, and calcium fluorosilicate glass Liquid: urethane dimethacrylate, tricyclodecane dimethanol dimethacrylate, tetramethyl-xylylen diurethane dimethacrylate, polyethylene glycol 400 dimethacrylate, Ivocerin, and hydroxyperoxide
Surefil One	Powder: silanated aluminum-phosphorus-strontium-sodium-fluoro-silicate glass, dispersed silicon dioxide, ytterbium fluoride, and pigments Liquid: acrylic acid, polycarboxylic acid, bifunctional acrylate, self-cure initiator, camphorquinone, and stabilizer
